# Enrichment culture and identification of endophytic methanotrophs isolated from peatland plants

**DOI:** 10.1007/s12223-017-0508-9

**Published:** 2017-03-09

**Authors:** Zofia Stępniewska, Weronika Goraj, Agnieszka Kuźniar, Natalia Łopacka, Magdalena Małysza

**Affiliations:** Department of Biochemistry and Environmental Chemistry, Institute of Biotechnology, The John Paul II Catholic University of Lublin, Konstantynow 1I, 20-708 Lublin, Poland

## Abstract

Aerobic methane-oxidizing bacteria (MOB) are an environmentally significant group of microorganisms due to their role in the global carbon cycle. Research conducted over the past few decades has increased the interest in discovering novel genera of methane-degrading bacteria, which efficiently utilize methane and decrease the global warming effect. Moreover, methanotrophs have more promising applications in environmental bioengineering, biotechnology, and pharmacy. The investigations were undertaken to recognize the variety of endophytic methanotrophic bacteria associated with *Carex nigra*, *Vaccinium oxycoccus*, and *Eriophorum vaginatum* originating from Moszne peatland (East Poland). Methanotrophic bacteria were isolated from plants by adding sterile fragments of different parts of plants (roots and stems) to agar mineral medium (nitrate mineral salts (NMS)) and incubated at different methane values (1–20% CH4). Single colonies were streaked on new NMS agar media and, after incubation, transferred to liquid NMS medium. Bacterial growth dynamics in the culture solution was studied by optical density—OD600 and methane consumption. Changes in the methane concentration during incubation were controlled by the gas chromatography technique. Characterization of methanotrophs was made by fluorescence in situ hybridization (FISH) with Mg705 and Mg84 for type I methanotrophs and Ma450 for type II methanotrophs. Identification of endophytes was performed after 16S ribosomal RNA (rRNA) and mmoX gene amplification. Our study confirmed the presence of both types of methanotrophic bacteria (types I and II) with the predominance of type I methanotrophs. Among cultivable methanotrophs, there were different strains of the genus *Methylomonas* and *Methylosinus*. Furthermore, we determined the potential of the examined bacteria for methane oxidation, which ranged from 0.463 ± 0.067 to 5.928 ± 0.169 μmol/L CH4/mL/day.

## Introduction

Cultivation of methanotrophs was started in 1906 by Söhngen, who isolated *Bacillus methanicus* (now known as *Methylomonas methanica*) (Söhngen 1906). Since that time, the number and diversity of cultured methanotrophs have significantly increased. The crucial studies on cultivation of methanotrophs were performed by Whittenbury et al. (1970), who isolated more than 100 strains of these bacteria from various environments. The two mineral media containing nitrate or ammonium mineral salts (NMS and AMS) optimized by Whittenbury et al. are still widely used. So far, methanotrophic bacteria have been detected in different types of ecosystems both natural: soils, deserts, landfills, tundra, wetlands, rice paddies, sediments, lakes, marine (Hanson and Hanson 1996), as well as in the atmosphere (Santl-Temkiv et al. 2013), coal mines (Stępniewska et al. 2006), and mercury mines (Baesman et al. 2015) as well as engineered ecosystems, e.g., waste water treatment plant (Ho et al. 2013).

**Fig. 1:**
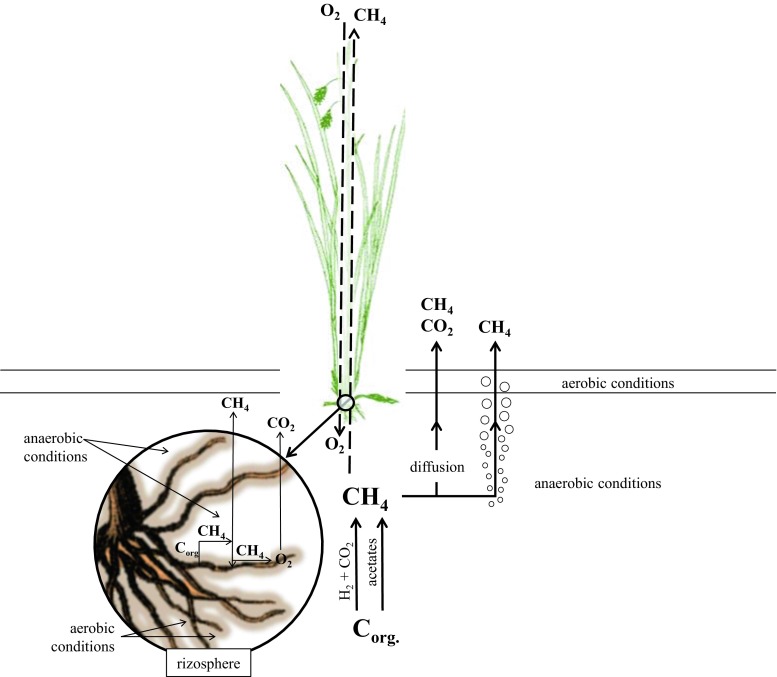
CH_4_ production, transport, and consumption in the soil-plant-atmosphere system (Kiene 1991, Le Mer and Roger 2001 with own modification)

Wetlands are the main natural source of methane. This emission is the result of the balance between methanogenesis and methanotrophic processes and is actively affected by the composition of wetland plants, which can influence CH_4_ production, consumption, and transport in the soil. Studies on this phenomenon indicated a significant role of methanotrophic bacteria in CH_4_ emissions, both the free-living microorganisms in the rhizosphere attached to the root surface in the form of a biofilm (rhizoplane) and those living inside host tissues (endophytes) and colonizing parts of plants (Raghoebarsing et al. 2005; Liebner et al. 2011; Stępniewska et al. 2013; Stępniewska and Kuźniar 2013; Putkinen et al. 2014). The mechanism of the association of methanotrophs with peat vascular plants is poorly recognized and is currently the subject of studies undertaken by many researchers throughout the world (Fig. 1).

During the first step in methane oxidation to CO_2_, an important role is played by methane monooxygenase (MMO), which catalyzes the conversion of methane to methanol (Hanson and Hanson 1996). Methane monooxygenase occurs in two forms: membrane-bound, particulate (pMMO) and cytoplasmic soluble (sMMO). sMMO is responsible for the catalysis of oxidation of some hydrocarbons (saturated, unsaturated, linear, branched, and cyclic hydrocarbons, single- and double-ring aromatics, heterocycles, halogenated alkenes, and ethers (Dunfield et al. 1999)). All aerobic methanotrophs contain one or both MMOs. pMMO is coded by a *pmo*CAB operon and sMMO by genes including *mmo*XYBZDC. The *pmo*A and *mmo*X genes are usually used as functional markers for methanotrophic bacteria. Detection of *pmo*A or *mmo*X gene sequences from new isolates combined with determination of the 16S ribosomal RNA (rRNA) gene sequence is a necessary requirement for identification of methanotrophs (Dedysh and Dunfield 2014). *mmo*X is characteristic of most type II methanotrophs as well as in members of the genus *Methylococcus* and some *Methylomonas* strains (Shigematsu et al. 1999), *Methylovulum miyakonense* HT12 (Iguchi et al. 2011), but not in most of the other type I methanotrophs (Hanson and Hanson 1996).

Methanotrophs are multifunctional, harmless bacteria with promising applications in environmental bioengineering of methane removal and biodegradation of toxic compounds. The biodiversity of the methanotroph habitat especially with an established presence of sMMO gives many opportunities for application of these unique bacteria (Sullivan et al. 1998; Pandey et al. 2014).

## Material and methods

### Plant samples

Plant materials were collected from the Moszne Lake area located in the northwestern part of the Poleski National Park (51° 23′ N, 23° 63′ E), situated in the province of Lublin, in the Polish part of Polesie, where vegetation was dominated by *Sphagnum magellanicum*, *Sphagnum fuscum*, *Sphagnum fallax*, *Aulacomium palustre*, *Polytrichum strictum*, and *Drosera rotundifolia* with dense colonies of *Vaccinium* species, *Eriophorum vaginatum*, *Eriophorum angustifolium*, *Carex rostrate*, *Carex nigra*, and *Carex gracilis.*

### Methanotrophic bacteria isolation and culture

Methanotrophic bacteria were isolated from plants *C. nigra* (*C*), *Vaccinium oxycoccus* (*V*), and *E. vaginatum* (*E*) by adding sterile fragments of each part of plant: roots (*R*) and stems (*S*) to agar mineral medium (NMS) (Whittenbury et al. 1970) and incubated in methane atmosphere 10% (*v*/*v*) CH_4_. Fragments of plants were sterilized by sequential immersion in 70% (*v*/*v*) ethanol for 5 min, and 0.9% (*w*/*v*) sodium hypochlorite solution for 20 min, and then surface-sterilized samples were washed in sterile water for three times to remove surface sterilization agents. Single colonies were streaked on new NMS agar media and, after incubation, transferred to liquid NMS medium. The cultures were incubated at 30 °C and 180 rpm (Innova 42R, New Brunswick Scientific, USA) under atmospheric conditions supplemented with 1, 5, 10, and 20% (*v*/*v*) CH_4._

Bacterial growth dynamics was studied by absorbance and methane consumption. Changes in the methane concentrations were controlled during incubation by the gas chromatography technique. The headspace concentrations of gases (CH_4_, CO_2_, O_2_, N_2_) were analyzed by a gas chromatograph Shimadzu GC 2010 (Japan) equipped with a flame ionization detector and a thermal conductivity detector, after CH_4_, CO_2_, O_2_, and N_2_ calibrations. Bacterial growth dynamics was assessed by measuring absorbance at 600 nm (*A*_600_) (UV1800, Shimadzu, Japan).

### Calculation and statistical analysis

The methanotrophic activity of cultures was calculated from the slope of the regression line of the measured CH_4_ concentration vs. time. Adjustments were made with *r* ^2^ ≤ 0.95 with Sigma Plot 10.0 software (USA) and written as micromole per liter CH_4_ per milliliter of culture per day. MMO enzyme activity was characterized by a curve of initial velocity vs. substrate concentration, referred to as a Michaelis-Menten plot, with an inset Lineweaver-Burk plot. The maximum specific methane oxidation rate (Vmax) and saturation constant (Km) were determined using GraphPad Prism 6 software (USA).

### DNA isolation and amplification

Bacterial DNA was isolated from the cultures with the method of Sambrook with own modifications. (Sambrook 1989). Cultured cells were harvested by centrifugation at 17500*g* for 5 min and subjected to lysis using 5 mol/L guanidine thiocyanate, 100 mmol/L EDTA, and 0.5% sarcosyl (pH 8.0). DNA was purified by extraction with ice-cold 7.5 mol/L ammonium acetate (10 min) and, subsequently, using a chloroform/3-methyl-1-butanol (24:1, *v*/*v*) mixture (1 min). The two-phase mixture was centrifuged at 17500*g* for 10 min. The upper layer was collected into a new tube. DNA was precipitated at −20 °C with 0.8 volumes of 2-propanol for 1 h. The pellet was separated by centrifugation at 17,500*g* for 30 min, rinsed five times with 70% (*v*/*v*) ethanol, dried under vacuum (RVC 2_18, Christ, Germany), and resuspended in 30 mL of ultrapure, DNAse-free water. The purity and concentration of the isolates were evaluated spectrophotometrically (UV_1800, Shimadzu, Japan). 16S rRNA genes were amplified from enrichment culture DNA using primers 27f (AGAGTTTGATCMTGGCTCAG) and 1492r (TACGGYTACCTTGTTACGACTT), (DeLong 1992). The presence of the *mmo*X gene in the isolates encoding sMMO, which are key enzymes in the bacterial methane metabolism pathway, was used for authentication of the isolates. *mmo*X gene was detected due to the oxidation of methane in sMMO is critical for understanding the microbial applications of C-H activation in one-carbon substrates and limited diversity of sMMO-containing methanotrophs (Lee 2016). The presence of genes in the isolates was detected by partial amplification of the genes using specific primers. The following primer pairs were used for *mmo*X (*mmo*X1 CGGTCCGC TGTGGAAGGGCATGAAGCGCGT and *mmo*X2 - GGCTCGACCTTGAACTTGGAGCCA TACTCG) (Miguez et al. 1997). The PCR reaction mixture (50 μL) contained PCR Master Mix (2×): 0.05 U/μL *Taq*DNA polymerase, reaction buffer, 4 mmol/L MgCl_2_ and 0.4 mmol/L of each dNTP, (Thermo Scientific); forward and reverse primers at 0.1 mmol/L and 3 μL of template DNA. PCR amplification was performed in a TProfessional gradient system thermocycler (Biometria, Germany) using the following conditions: initial denaturation at 95 °C (1 min); 30 cycles consisting of denaturation 95 °C (0.30 min), annealing 55 °C (1 min) for 16S rRNA, 62.5 °C (0.55 min) for *mmo*X primers; extension 72 °C (1 min); and final extension 72 °C (5 min). Isolated DNA and PCR products were analyzed by gel electrophoresis on MiniSubR Cell GT (Bio_Rad Laboratories Ltd., UK), stained with ethidium bromide, and visualized in UV light in the Red™ Imaging System (Alpha Innotech, USA).

### DNA sequence analysis

The identification of the cultured plant endophytes was performed after PCR product sequencing (Genomed S.A., Poland) and compared with sequences stored in NCBI (USA) using the BLASTN algorithm.

### Nucleotide sequence

The 16S rRNA gene sequences of the methanotrophic communities examined in this study were deposited in GenBank databases under accession numbers KT860052, KT860053, KT860054, KT860055, and KT860056 numbers. Moreover, as a result of our investigation, we added *mmo*X gene sequences as KT962955, KT962956, KT962957, KT962958, and KT962959 to the GenBank databases (Table 1).

**Table 1:** Sequences of the endophytes deposited in GenBank databases

Accession numbers	Gen	Isolation_source	Isolate
KT860052	16S rRNA	*C. nigra* stream	*Methylomonas* sp. enrichment culture PPN CS1
KT860053	16S rRNA	*C. nigra* root	Bacterium enrichment culture PPN CR2
KT860054	16S rRNA	*V. oxycoccus* stream	Bacterium enrichment culture PPN VS1
KT860055	16S rRNA	*E. vaginatum* root	*Methylomonas* sp. enrichment culture PPN ER1
KT860056	16S rRNA	*E. vaginatum* root	Bacterium enrichment culture PPN ER2
KT962955	*mmo*X	*C. nigra* stream	*Methylosinus* sp. PPN CS2
KT962956	*mmo*X	*C. nigra* stream	*Methylomonas* sp. PPN CR1
KT962958	*mmo*X	*E. vaginatum* stream	*Methylosinus* sp. PPN ER1
KT962959	*mmo*X	*E. vaginatum* stream	*Methylosinus* sp. PPN ER2

### FISH from endophyte cultures

FISH was performed according to Eller et al. (2001) with minor modifications using cells growing in the logarithmic phase. Cells were harvested by centrifugation and resuspended in 0.5 mL of phosphate-buffered saline (PBS). The suspensions were then mixed with 1 mL of 4% (*w*/*v*) paraformaldehyde and fixed for 3 h. The fixed cells were collected by centrifugation (6000*g* for 1 min) and washed twice with PBS. The resulting pellet was resuspended in 50% ethanol in PBS and stored at −20 °C until use. The hybridization was carried out on slides where 15 μL of fixed cells suspensions were transferred and left to dry. Dehydration was performed by washing the slides in 50, 80, and 96% (*v*/*v*) ethanol for 3 min each. A 50-mL polypropylene falcon tube containing a slip of filter paper soaked in hybridization buffer was used as a hybridization chamber. Ten-microliter hybridization buffer (1 mol/L Tris-HCl (pH 8.0), 5 mol/L NaCl, 10% sodium dodecyl sulfate and 20% formamide), and 1 μL of a fluorescent probe solution was placed on each spot of the fixed cells. The specificity of the probes applied is presented in Table 2. The chamber was incubated for 90 min at the hybridization temperature (47 °C). Then, the slides were washed at the hybridization temperature for 15 min in washing buffer (1 mol/L Tris-HCl, 0.5 mol/L EDTA, and 5 mol/L NaCl) and rinsed twice with distilled water. The slides were air-dried and stained with Vectashield Mounting medium containing DNA-staining DAPI (Vector Laboratories, USA). The slides were analyzed using fluorescence with a Nikon Eclipse 80i microscope. The pictures were taken with a Digital Sight camera (Nikon) and processed with software provided by the manufacturer.

**Table 2:** Characterization of probes used in FISH

Probe	Mg84	Mg705	Ma450
Specificity	Type I	Type I	Type II
Sequence 5′–3′	CCACTCGTCAGCGCCCGA	CTGGTGTTCCTTCAGATC	ATCCAGGTACCGTCATTATC
Target gen	16S rRNA	16S rRNA	16S rRNA
Fluorescent dye 5′	Cy3	Fluoresceine	Cy5

## Results

### Endophyte enrichment culture growth

**Fig. 2:**
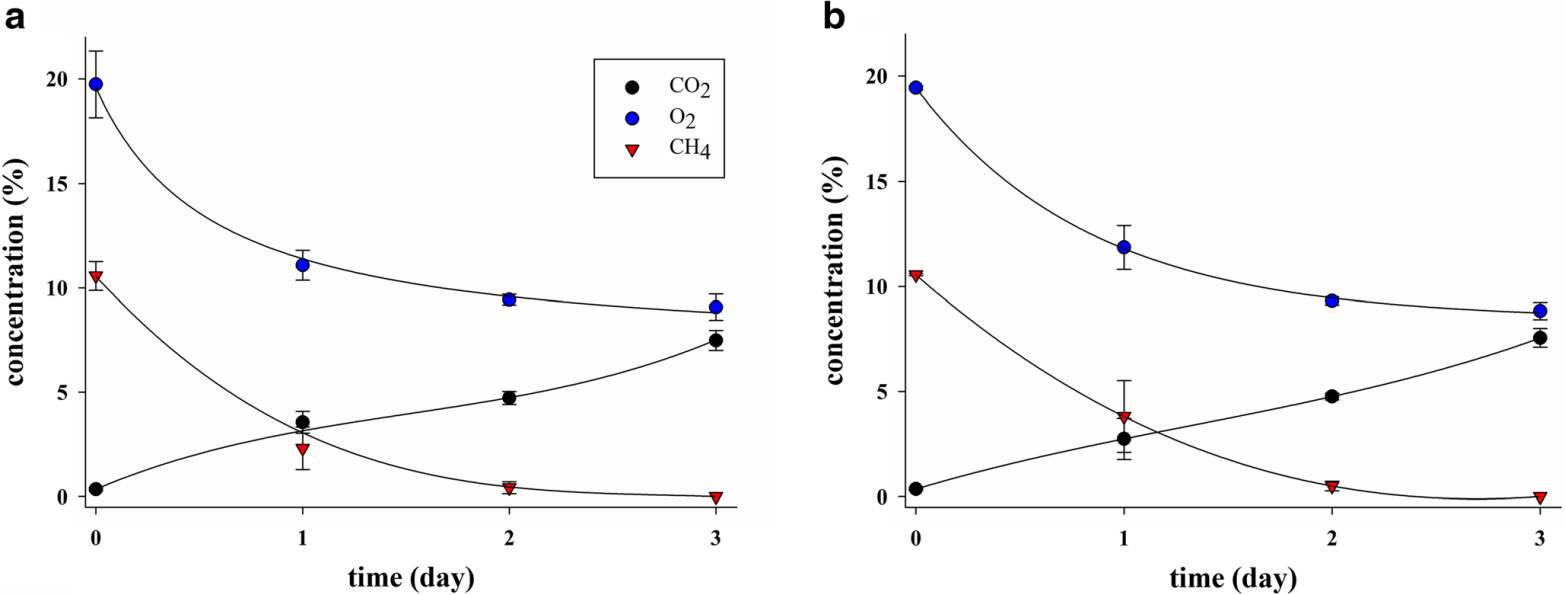
Dynamics of the tested gases during the growth of the methanotrophic community isolated from *Carex* sp. roots (**a**) and *Carex* sp. stems (**b**) under 10% CH_4_ in the initial headspace

Endophytes of *C. nigra*, *V. oxycoccus*, and *E. vaginatum* were grown on 5, 10, and 20% of CH_4_ at 30 °C. Cultivations of all methane-oxidizing bacteria (MOB) consortia were characterized by the dynamics of the gaseous phase. A steady decrease in the methane and oxygen concentration was shown, as well as accumulation of carbon dioxide (Fig. 2). The CH_4_ consumption in the all investigated enrichment culture showed a decrease in the CH_4_ concentration during 4–6 days of incubation.

**Fig. 3:**
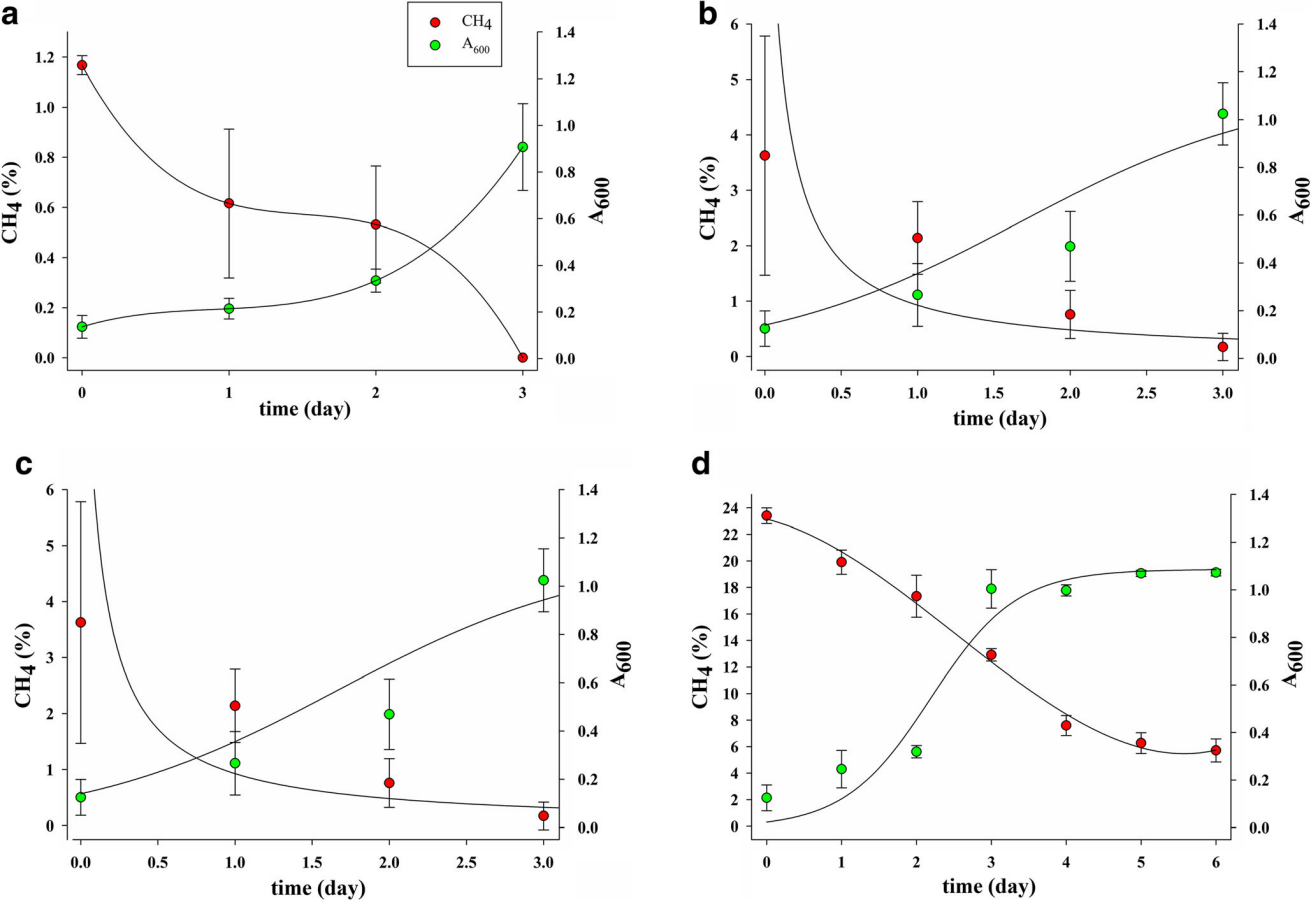
Growth curves of methanotrophic consortium cultures isolated from *Carex* sp. at different methane concentrations (**a** 1%; **b** 5%; **c** 10%; **d** 20%)

Rapid growth of all investigated MOB consortia was observed; it was reflected by an increase absorbance in the range from about 0.1 to 0.8–1.8 after 3 to 6 days of cultivation, which was accompanied by a decline in the CH_4_ mixing ratio in the headspace, while no growth was observed in the same medium in the absence of methane. The complete consumption of CH_4_ by methanotrophic consortium cultures after 3 days under 1–10% CH_4_ was demonstrated. At the highest substrate concentration (20% CH_4_), the presence of 6% of methane was shown after 6 days of cultivation (Fig. 3).

### Methanotrophic activity of the endophytes

**Fig. 4:**
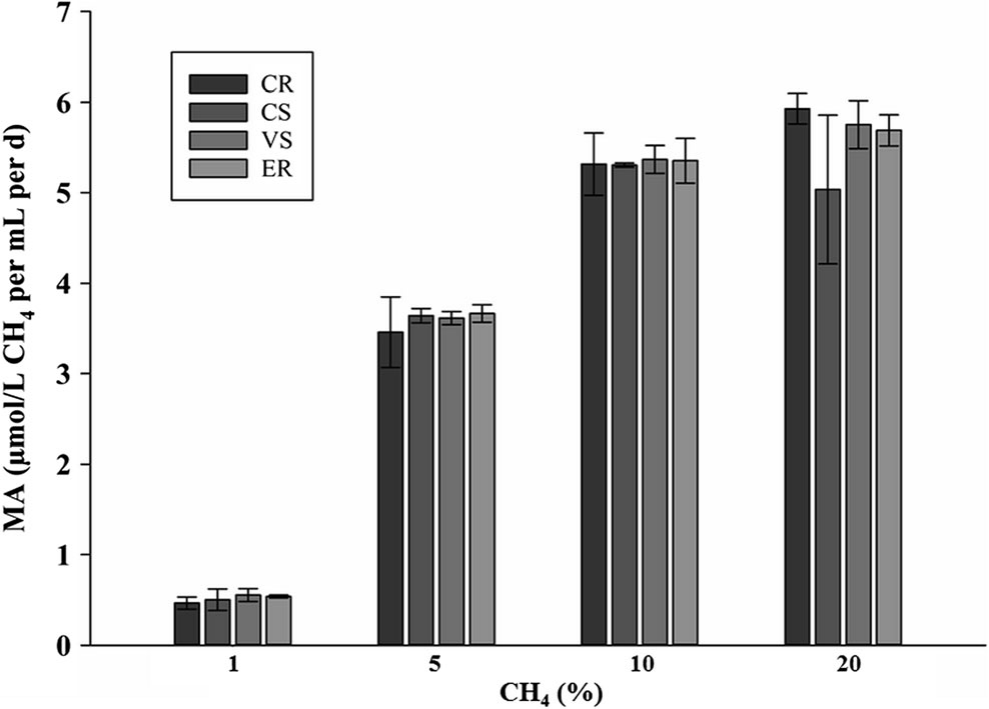
Methanotrophic activity of the tested endophytic consortium (CR = *C. nigra* root, CS = *C. nigra* stem, ER = *E. vaginatum* root, VS = *V. oxycoccus* stem) enrichment cultures at different methane concentration

On the basis of the consumption of methane during culture, the methanotrophic activity of the endophytes was determined. A clear decrease in the CH_4_ concentration tested in the headspace was shown after 4–6 days of incubation. Methane consumption by the consortium isolated from the roots of wetland plants ranged from 0.463 ± 0.067 to 5.928 ± 0.169 μmol/L CH_4_/mL/day and from the stems of these plants from 0.499 ± 0.119 to 5.305 ± 0.022 μmol/L CH_4_/mL/day, depending on the initial CH_4_ concentration (Fig. 4). The dependence of the methanotrophic activity of isolated methanotrophs was significantly related to the substrate concentration (*p* < 0.0001) and did not differ significantly between the plant species.

### Kinetic parameters of CH_4_ oxidation

**Fig. 5:**
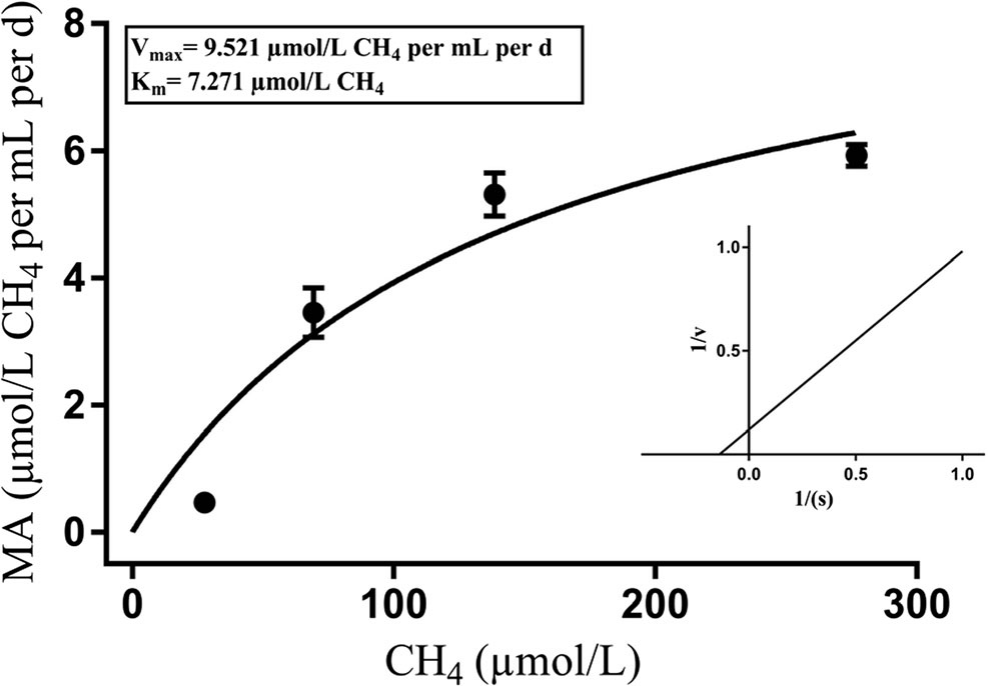
Example of Michaelis-Menten saturation curves of CH_4_ oxidation in CR enrichment cultures

**Fig. 6:**
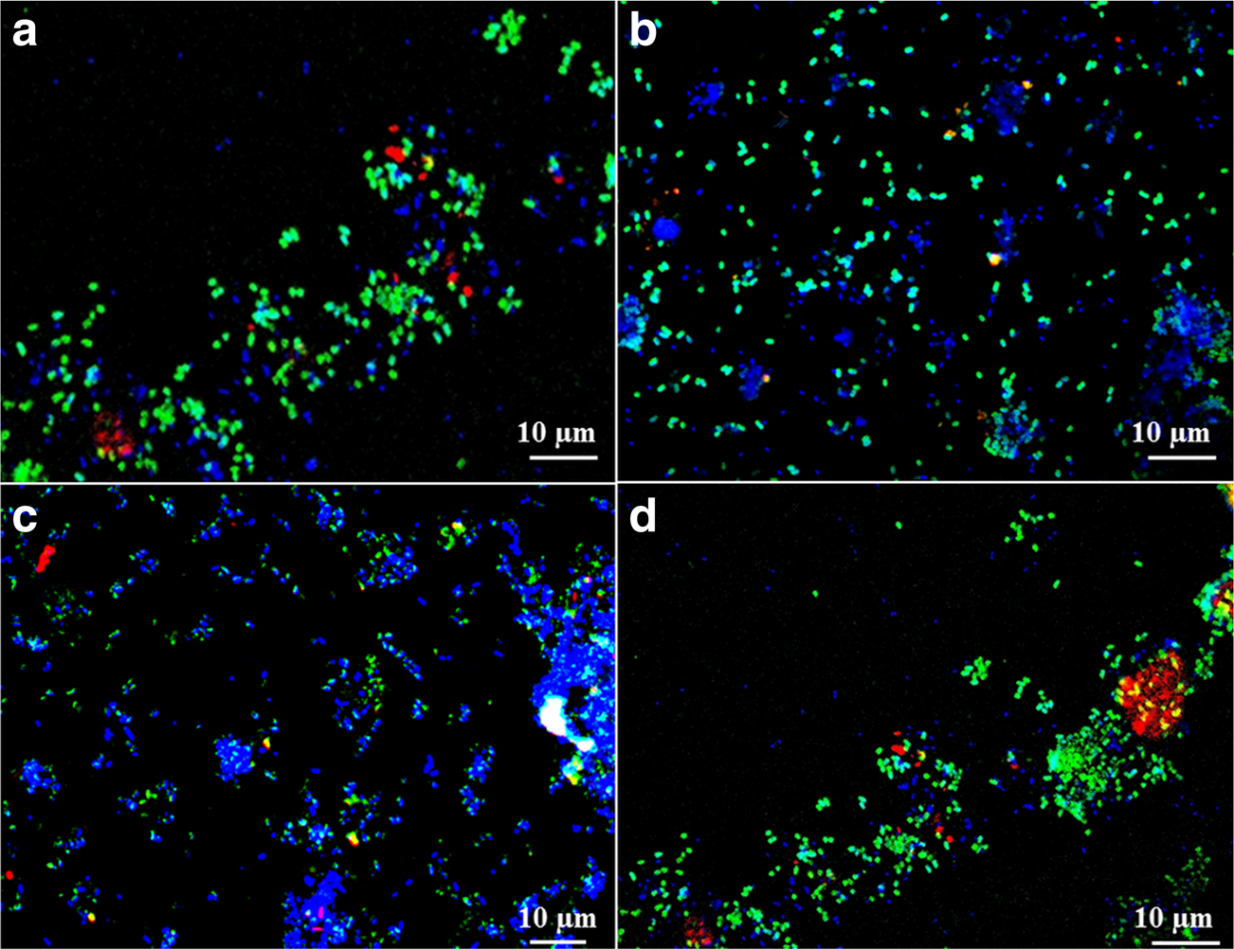
Fluorescent in situ hybridization of the consortium from *C. nigra* roots (**a**) and stems (**b**), *V. oxycoccus* stems (**c**) and *E. vaginatum* roots after hybridization with Mg84 and Mg705 probes (type I) *green colored*, Ma450 probe (type II) *red colored*, and DAPI staining probes (non-methanotrophic) *blue colored*

The CH_4_ oxidation rates were written correctly in the substrate saturation curves as a function of initial methane concentrations (Fig. 5) and showed typical Michaelis-Menten characteristics. Lineweaver-Burk plots based on regression of 1/*V* vs. 1/*S* are presented as insets in Fig. 6. The apparent Km and Vmax values obtained for the tested enrichment culture are summarized in Table 3. The methane oxidation by the methanotrophic consortium enrichment cultures followed the Michaelis-Menten mechanism. In a majority of the analyzed cases, the specific methane oxidation rates increased in parallel with the initial methane concentration, but only the CS consortium enrichment culture achieved the maximum rate at 10% methane 5.033 ± 0.823 μmol/L CH_4_/mL/day. Based on the Lineweaver-Burk plot (Fig. 5), Vmax, and Km were calculated in the range from 9.521 ± 1.280 to 7.539 ± 1.115 μmol/L CH_4_/mL/day and from 142.3 ± 40.69 to 95.42 ± 34.96 μmol/L CH_4_/mL/day, respectively (Table 3).

**Table 3:** Kinetic parameters of CH_4_ oxidation in tested enrichment cultures

Endophytes cultures	Vmax (μmol/L CH_4_/mL/day)	Km (μmol/L CH_4_)
CR (*C. nigra* root)	9.521 ± 1.280	142.3 ± 40.69
CS (*C. nigra* stream)	7.539 ± 1.115	95.42 ± 34.96
VS (*V. oxycoccus* stream)	8.705 ± 0.98	117.6 ± 30.25
ER (*E. vaginatum* root)	8.891 ± 1.094	122.1 ± 33.84

± Std. Error

### Fluorescence in situ hybridization

Characterization of MOB was performed by fluorescence in situ hybridization (FISH) with Mg705 and Mg84 for type I methanotrophs and Ma450 for type II methanotrophs. Microscopic observations confirmed the presence of methanotrophic microorganisms isolated from the plants and grown on NMS medium. The combination of oligonucleotide probes Mg84 and Mg705 with Ma450 allowed parallel detection of type I and type II methanotrophs with the predominance of type I. Type I cells (shown as green) were short 1.5–3-μm-long rods and were all similar in size and shape. Type II methanotrophs were less numerous but more diverse. These bacterial cells represented at least two distinct morphologies: straight (1–2 μm long) and irregular, curved bacilli (0.5–1 μm). Simultaneously, the staining with DAPI revealed that methanotrophic bacteria were present in the community with non-methanotrophic cells which are almost as numerous as the methanotrophic cells (Fig. 6).

### Identification of cultured endophytic methanotrophs

Analysis of the partial 16S rRNA gene sequences of the isolated MOB showed that they clustered closely with homologous sequences from different strains of the genus *Methylomonas* and numerous uncultured bacteria (Table 4).

**Table 4:** Identification of cultured endophytic methanotrophs based on 16S rRNA gene

Isolate	Identity	Plant species	Ident (%)	Accession number
*Methylomonas* sp. enrichment culture PPN CS1	*Methylomonas* sp. BG3 *Methylomonas* sp. DH-1 *Methylomonas koyamae* strain Fw12E-Y	*C. nigra*	99 98 98	KJ081955.1 KT799650.1 NR_113033.1
Bacterium enrichment culture PPN CR2	*Methylomonas* sp. DH-1 *Methylomonas* sp. R-45381 *Methylomonas* sp. R-45378	*C. nigra*	93 93 93	KT799650.1 FR798966.1 FR798963.1
Bacterium enrichment culture PPN VS1	*Methylomonas* sp. BG3 *Methylomonas* sp. R-45383 Uncultured bacterium clone DS-10	*V. oxycoccus*	92 91 91	KJ081955.1 FR798968.1 KC424661.1
*Methylomonas* sp. enrichment culture PPN ER1	*Methylomonas* sp. BG3 *Methylomonas* sp. B2Z *Methylomonas* sp. R4F	*E. vaginatum*	97 96 96	KJ081955.1 AB683103.1 AB636300.1
Bacterium enrichment culture PPN ER2	Uncultured bacterium clone SHCB0889 Uncultured bacterium clone c5LKS3 Uncultured bacterium clone S130	*E. vaginatum*	95 95 95	JN698029.1 AM086104.1 KR095256.1

It was found that sequences obtained from the endophyte enrichment culture with *C. nigra* stream (PPN CS1) were related with 99–98% identity to *Methylomonas* sp. [KJ081955.1, KT799650.1, NR_113033.1]. Isolate of *C. nigra* root (PPN CR2) demonstrated affiliation to bacteria but only 93% identity to *Methylomonas* sp. [KT799650.1, FR798966.1, FR798963.1]. The sequences of the methanotrophic microbial consortium isolated from *V. oxycoccus* (PPN VS1) showed affiliation to bacteria with 8–9% divergence from *Methylomonas* sp. [KJ081955.1, FR798968.1] and uncultured bacterium clone DS-10 [KC424661.1]. The population isolated from *E. vaginatum* (PPN E1) was related with 97–96% identity to *Methylomonas* sp. [KJ081955.1, AB683103.1, AB636300.1]. In the PPN ER2 isolate, we identified 95% similarity mainly to the uncultured bacterium (JN698029.1, AM086104.1, KR095256.1). Molecular identification on the basis of *mmoX* revealed high similarity of the methanotrophs cultured on the NMS medium to the genera *Methylosinus* and *Methylomonas* (Table 5)*.*

**Table 5:** Identification of cultured endophytic methanotrophs based on *mmo*X gene

Isolate	Identity	Plant species	Ident (%)	Accession number
*Methylosinus* sp. PPN CS1	*Methylosinus trichosporium* IMET 10561 *Methylomonas* sp. GYJ3 *Methylosinus trichosporium* 39/3	*C. nigra*	98 97 97	AJ458518.1 DQ149127.1 AJ458514.1
*Methylomonas* sp. PPN CR2	*Methylomonas* sp. GYJ3 *Methylosinus trichosporium* IMET 10561 *Methylosinus trichosporium* IMV 3011	*C. nigra*	97 97 97	DQ149127.1 AJ458518.1 DQ149126.2
*Methylosinus* sp. PPN VS1	*Methylosinus trichosporium* SC10 *Methylosinus trichosporium* 60 Uncultured bacterium clone RFSmmoX44	*V. oxycoccus*	97 97 95	AJ458524.1 AJ458519.1 KM924806.1
*Methylosinus* sp. PPN ER1	*Methylosinus trichosporium* IMET 10561 *Methylosinus trichosporium* strain 39/3 *Methylomonas* sp. GYJ3	*E. vaginatum*	98 97 97	AJ458518.1 AJ458514.1 DQ149127.1
*Methylosinus* sp. PPN ER2	*Methylosinus trichosporium* IMET 10561 *Methylosinus trichosporium* strain KS18 *Methylosinus trichosporium* strain SC10	*E. vaginatum*	98 97 97	AJ458518.1 AJ458530.1 AJ458524.1

## Discussion

It was confirmed that methanotrophic bacteria have great potential as microbial sinks for the greenhouse gas methane as well as for industry in different biotechnological solutions (Semrau et al. 2010). However, their application in biotechnology is limited by the number of suitable strains readily accessible, which were not specifically isolated for this purpose, and the lack of the necessary properties for efficient use on an industrial scale (Jiang et al. 2010). Among the disadvantages, the relatively slow growth rate of the available strains may be mentioned (Hoefman et al. 2010). In this context, searching for new methanotroph strains and investigation of methanotrophic bacterial communities takes on a new meaning.

The endophytic bacterial community isolated in our study from vascular peatland plants *C. nigra* (PPN CS1) and *E. vaginatum* (PPN ER1) are closely related to *Methylomonas* sp. belonging to type Ia methanotrophs, known as fast-growing MOB with a short generation time of 3.5 h (Whittenbury et al. 1970). Type II MOB is generally known to grow more slowly, with generation times ranging from 5 h up to several days (Whittenbury et al. 1970; Dedysh et al. 2000, 2002; Vorobev et al. 2010). The investigated methanotrophic consortium cultures were characterized by rather rapid growth, reaching absorbance 0.8–1.8 after 3–6 days of cultivation, depending on the initial CH_4_ concentration. As reported by literature, methanotrophs possessing pMMO show good growth with *A*_600_ reaching 0.8–1.5 within 3–6 days. By contrast, methanotrophs with only sMMO may require up to 2–3 weeks until the cultures reaches *A*_600_ of 0.2–0.3 (Dedysh and Dunfield 2014). The fast growth rate and lower initial cell numbers in combination shows typical R-type life strategy of the methanotrophs, investing in reproduction (Steenbergh et al. 2010). Methanotrophic enrichment culture isolated from *C. nigra* (PPN CS2), *V. oxycoccus* (PPN VS1), and *E. vaginatum* (PPN ER2) revealed 16S rRNA gene sequence similarities of less than 98% which does not allow to determination of the genus.

Methanotrophs associated with *C. nigra* stream were closely related to the strains found previously in waterlogged conditions such as wastewater (KJ081955.1), waste sludge (KT799650.1) and floodwater of paddy fields (NR_113033.1). Bacteria similar to these inhabiting *C. nigra* roots were found in wetlands (FR798966.1, FR798963.1). *V. oxycoccus* stream endophytes were similar to bacteria isolated from waste water (KJ081955.1) and wetland (FR798968.1) and to uncultured bacteria from lake sediments. The endophytes of *E. vaginatum* roots were similar to bacteria isolated from wastewater (KJ081955.1), root of *Acorus calamus* var*. angustatus* (AB683103.1), rice root (AB636300.1), and numerous uncultured bacteria e.g. isolated from the rice root endosphere (JN698029.1), profundal lake sediment (AM086104.1), and *Typha* rhizosphere in wetlands of the Yongding River (KR095256.1) (Table 3). PPN CR2, PPN VS1, and PPN ER2 isolates may be particularly important because the nearest phylogenetic neighbor indicates the presence of a novel species. The full characterization and description of novel species which will be made in the future will complement these studies.

Stępniewska and Kuźniar (2014)) cultured endophytic methanotrophs isolated from different *Sphagnum* species originating from Moszne peatbog. The growth of this endophytic population was characterized by an increase in absorbance in the range from 0.3 to 2.0 after 12–13 days of incubation. Among cultivable type I methanotrophs, Stepniewska and Kuzniar (2014) identified different strains of the genus *Methylomonas*, whereas type II methanotrophs were represented by cultured strains belonging to the genera *Methylocystis* and *Methylosinus*. Methane consumption by the isolated consortiums ranged from 0.463 ± 0.067 to 5.928 ± 0.169 μmol/L CH_4_/mL/day. Methanotrophs isolated from different *Sphagnum* species and cultured at the same conditions were characterized by a lower MA, reaching maximum of 4.7 μmol/L CH_4_/mL/day in the isolated populations from *S. magellanicum* (Stepniewska and Kuzniar 2014).

sMMO is present in most type II methanotrophs and in members of the genus *Methylococcus* and *Methylomonas* (Shigematsu et al. 1999). In this study, based on the 16S rRNA, gene *Methylomonas* sp. affiliation was found, but amplification of the *mmo*X gene and DNA sequencing indicates the presence of the genus *Methylosinus* and *Methylomonas.* This data suggest that analysis of sequence of *mmo*X allowed noting a closer affinity to the GenBank database sequences and classification of isolates to *Methylosinus* sp. The limited diversity of sMMO may be related to constraints on the genetic diversity of this enzyme due to the conservation of the enzyme function, or may simply be found because strategies used to isolate methanotrophs by culturing or detection of *mmo*X by PCR are yet to reveal the true diversity of sMMO-containing methanotrophs.

Methanotrophic bacteria of the genus *Methylosinus* and *Methylomonas* are widespread in different environments such as: peatland soil (Szafranek-Nakonieczna et al. 2012; Danilova et al. 2013; Esson et al. 2016), peatland plants (Raghoebarsing et al. 2005; Iguchi et al. 2012; Stępniewska and Kuźniar 2014), lake sediment (Nercessian et al. 2005), paddy field (Ogiso et al. 2012), seawater (Boden et al. 2011), and coal mine (Wolińska et al. 2013). On the basis of these studies and the other data (e.g., Iguchi et al. 2012; Stępniewska and Kuźniar 2014), which indicate the presence of *Methylomonas* sp. and *Methylosinus* sp. associated with plants (mosses, herbaceous plants, woody plants), we can assume that these microorganisms may be particularly preferred by plants. The mechanism of this specific association requires further study. However, the coexistence of these bacteria with vascular plants can be justified by the ability to utilize methanol by *Methylomonas* sp. and *Methylosinus* sp. (Gayazov et al. 1990; Xin et al. 2010; Danilova et al. 2013). It is known that based on plant structure and metabolic properties, particularly the demethylation of pectin in the primary cell walls, plants produce methanol (Galbally and Kirstine 2002) and plant-associated methylobacteria was confirmed (Kutschera 2007).

Endophytes isolated from *Sphagnum* sp. and vascular peatland plants investigated in this study were characterized as communities. Many colonies that appear on solid mineral media after incubation in the presence of the methane belong to non-methanotrophs. Typical examples of such non-methanotrophic bacteria are members of the genera *Burkholderia* and *Bradyrhizobium* (Dedysh and Dunfield 2014). Microorganisms from communities interacting at multi-trophic levels (Naeem and Li 1997; Naeem et al. 2000) are grouped into autotrophs (primary producers) and heterotrophs (decomposers). This kind of relationships may be observed in methane driven ecosystems, where the methanotroph can be considered as a primary producer and interacts with heterotrophs (Hutchens et al. 2004; Iguchi et al. 2011; van Duinen et al. 2013; Agasild et al. 2014). Ho et al. (2014) showed that methanotrophic activity was positively correlated with heterotroph richness. Methanotrophs require associated microorganisms which can be important for growth and functioning (Hoefman et al. 2010).

This fact seems to be very important especially in connection with ecological significance of association MOB consortia with plants. Studies about *Sphagnum* sp. and endophytic methanotrophs such as: Raghoebarsing et al. (2005), Kip et al. (2010), Liebner et al. (2011), Stępniewska et al. (2013) and Stępniewska and Kuźniar (2014) showed that methane, being oxidized by methanotrophs to carbon dioxide, becomes an additional source of C to the plants. On the other hand the mosses provide oxygen and ecological niche for the methanotrophs. Recent studies Ho and Bodelier (2015) and Kox et al. (2016), emphasize that CH_4_–N cycle interactions were found in the methanotrophs associated with submerged mosses. The presence of a methanotrophic diazotrophs and fixation of N_2_ (diazotrophic activity) at a level of from 2 to 3 up to 35% of the N assimilated by *Sphagnum* sp. was demonstrated (Berg et al. 2013; Larmola et al. 2014). A similar interaction was observed in methanotrophic bacteria association with rice root (Minamisawa et al. 2016). These arguments testify to the fact that methanotrophs are an important component of the microflora of peat land.

Literature describes a wide range of methanotoph applications and is constantly looking for new efficient strains for biotechnology applications. The richness of non-methanotrophic bacteria associated with the endophyte community was confirmed by FISH analysis (Fig. 6). However, further studies are needed to determine whether the effect of the heterotroph richness is species specific and can be extrapolated to other methanotrophs.

In conclusion, the results of this study demonstrate that cultivable bacteria existing as endophytes of vascular plants belong to type I methanotrophs and are characterized by fast growth and relatively high methanotrophic activity. Moreover, we showed that the community composed of methanotrophs and non-methanotrophs has potential applications in biotechnology systems.
